# Whole-Genome Sequence of *Serratia symbiotica* Strain CWBI-2.3^T^, a Free-Living Symbiont of the Black Bean Aphid *Aphis fabae*

**DOI:** 10.1128/genomeA.00767-14

**Published:** 2014-08-21

**Authors:** Vincent Foray, Alina S. Grigorescu, Ahmed Sabri, Eric Haubruge, Georges Lognay, Frederic Francis, Marie-Laure Fauconnier, Thierry Hance, Philippe Thonart

**Affiliations:** a Catholic University of Louvain, Earth and Life Institute, Biodiversity Research Centre, Louvain-la-Neuve, Belgium; b University of Liège, Walloon Center of Industrial Biology, Liège, Belgium; c University of Liège, Gembloux Agro-Bio Tech, Functional and Evolutionary Entomology Unit, Gembloux, Belgium; d University of Liège, Gembloux Agro-Bio Tech, Laboratory of Analytical Chemistry, Gembloux, Belgium; e University of Liège, Gembloux Agro-Bio Tech, General and Organic Chemistry Unit, Gembloux, Belgium

## Abstract

The gammaproteobacterium *Serratia symbiotica* is one of the major secondary symbionts found in aphids. Here, we report the draft genome sequence of *S. symbiotica* strain CWBI-2.3^T^, previously isolated from the black bean aphid *Aphis fabae*. The 3.58-Mb genome sequence might provide new insights to understand the evolution of insect-microbe symbiosis.

## GENOME ANNOUNCEMENT

The bacterium *Serratia symbiotica* is described as a mutualistic inherited endosymbiont found in many aphid species (1). Two different phylogenetic clades have been reported for this species (2). On the one hand, clade A is composed of facultative endosymbionts infecting several aphid families. In the pea aphid *Acyrthosiphon pisum*, this facultative endosymbiont is associated with heat stress tolerance and parasite resistance (3, 4). On the other hand, the clade B members are restricted to aphids of the subfamily *Lachinae* and correspond to primary-like endosymbionts implicated in the synthesis of amino acids, like in the aphid *Cinara cedri* (5). Recently, *S. symbiotica* strain CWBI-2.3^T^ was isolated from *Aphis fabae* and cultivated on artificial rich medium (6), constituting the first symbiotic bacterium of aphids with a free-living capacity.

The complete genome of *S. symbiotica* strain CWBI-2.3^T^ was sequenced using the Pacific Biosciences RS sequencing technology (Pacific Biosciences, Menlo Park, CA, USA). A 10-kb library was prepared from sheared genomic DNA using a 10-kb template library preparation workflow. The library was sequenced on two single-molecule real-time (SMRT) cells, yielding >40× average genome coverage, with 64,305 reads with a mean read length of 3,187 bp. Assembly of the reads was done with the HGAP2 version 2.1 *de novo* assembly pipeline. Coding sequence prediction and automatic functional annotation were performed using the MicroScope platform (7).

The draft genome of *S. symbiotica* strain CWBI-2.3^T^ consists of 32 contigs, corresponding to 3,584,847 bp, with a G+C content of 52.08%. The genome contains 3,664 predicted protein-coding sequences, 74 tRNA genes, and 32 rRNAs (10 16S rRNAs, 12 23S rRNAs, and 10 5S rRNAs). It also includes the 203 single-copy genes conserved among gammaproteobacterial genomes (8).

The CWBI-2.3^T^ strain belongs to *S. symbiotica* clade A and is phylogenetically close to the *S. symbiotica* strain Tucson of the pea aphid *A. pisum*, based on a high nucleic acid identity for the 16S rRNA genes (99%). However, the CWBI-2.3^T^ strain has a different overall genomic structure and composition than those of the previously sequenced *S. symbiotica* strains. The total genome size of *S. symbiotica* strain CWBI-2.3^T^ is 0.80 and 1.82 Mb larger than the genomes of *S. symbiotica* strains Tucson and strain “*Cinara cedri*,” respectively. Furthermore, the CWBI-2.3^T^ strain conserved a larger repertoire of genes related to metabolism than did the two other strains. These results are consistent with the genomic erosion of host-dependent bacteria (9).

Most of facultative insect symbionts are uncultivable, precluding the development of genetic techniques used to understand host-symbiont interactions (10, 11). The genome of *S. symbiotica* strain CWBI-2.3^T^ reported here is the first genome of a symbiotic bacterium of aphids that is able to grow outside its host. It represents a missing link in the evolution of free living toward host-dependent mutualistic bacteria and provides the opportunity to carry on unique genomic comparative analyses and genetic modification experiments to investigate the bacterial symbiotic relationships in aphids.

### Nucleotide sequence accession numbers.

The complete genome sequences of *S. symbiotica* strain CWBI-2.3^T^ have been deposited in DDBJ/ENA/GenBank under accession numbers CCES01000001 to CCES01000032.

## References

[1] Burke GR Normark BB Favret C Moran NA 2009 Evolution and diversity of facultative symbionts from the aphid subfamily *Lachninae*. Appl. Environ. Microbiol. 75:5328–5335. 10.1128/AEM.00717-09 19542349 PMC2725466

[2] Lamelas A Pérez-Brocal V Gómez-Valero L Gosalbes MJ Moya A Latorre A 2008 Evolution of the secondary symbiont “Candidatus *Serratia symbiotica*” in aphid species of the subfamily *Lachninae*. Appl. Environ. Microbiol. 74:4236–4240. 10.1128/AEM.00022-08 18502932 PMC2446524

[3] Montllor CB Maxmen A Purcell AH 2002 Facultative bacterial endosymbionts benefit pea aphids *Acyrthosiphon pisum* under heat stress. Ecol. Entomol. 27:189–195. 10.1046/j.1365-2311.2002.00393.x

[4] Oliver KM Russell JA Moran NA Hunter MS 2003 Facultative bacterial symbionts in aphids confer resistance to parasitic wasps. Proc. Natl. Acad. Sci. U. S. A. 100:1803–1807. 10.1073/pnas.0335320100 12563031 PMC149914

[5] Lamelas A Gosalbes MJ Manzano-Marín A Peretó J Moya A Latorre A 2011 *Serratia symbiotica* from the aphid *Cinara cedri*: a missing link from facultative to obligate insect endosymbiont. PLoS Genet. 7:e1002357. 10.1371/journal.pgen.1002357 22102823 PMC3213167

[6] Sabri A Leroy P Haubruge É Hance T Frère I Destain J Thonart P 2011 Isolation, pure culture and characterization of *Serratia symbiotica* sp. nov., the R-type of secondary endosymbiont of the black bean aphid *Aphis fabae*. Int. J. Syst. Evol. Microbiol. 61:2081–2088. 10.1099/ijs.0.024133-0 20870890

[7] Vallenet D Engelen S Mornico D Cruveiller S Fleury L Lajus A Rouy Z Roche D Salvignol G Scarpelli C Médigue C 2009 MicroScope: a platform for microbial genome annotation and comparative genomics. Database 2009:bap021. 10.1093/database/bap021 PMC2790312 20157493

[8] Lerat E Daubin V Moran NA 2003 From gene trees to organismal phylogeny in prokaryotes: the case of the gamma-proteobacteria. PLoS Biol. 1:E19. 10.1371/journal.pbio.0000019 12975657 PMC193605

[9] Moran NA McCutcheon JP Nakabachi A 2008 Genomics and evolution of heritable bacterial symbionts. Annu. Rev. Genet. 42:165–190. 10.1146/annurev.genet.41.110306.130119 18983256

[10] Pontes MH Dale C 2006 Culture and manipulation of insect facultative symbionts. Trends Microbiol. 14:406–412. 10.1016/j.tim.2006.07.004 16875825

[11] Kikuchi Y 2009 Endosymbiotic bacteria in insects: their diversity and culturability. Microbes Environ. 24:195–204. 10.1264/jsme2.ME09140S 21566374

